# Family behavior theory-based intervention via mobile messaging to improve oral health of adolescents: study protocol for a cluster randomized controlled trial

**DOI:** 10.1186/s13063-022-06861-1

**Published:** 2022-11-16

**Authors:** Pei Liu, May Chun Mei Wong, Gillian Hiu Man Lee, Cynthia Kar Yung Yiu, Edward Chin Man Lo

**Affiliations:** 1 Applied Oral Sciences & Community Dental Care, Faculty of Dentistry, The University of Hong Kong, Hong Kong SAR, China; 2 Paediatric Dentistry, Faculty of Dentistry, The University of Hong Kong, Hong Kong SAR, China

**Keywords:** oral health, primary prevention, Health Belief Model, adolescents, mHealth, family-level intervention

## Abstract

**Background:**

Due to some unique physical, social and psychological features in the adolescent population, adolescents can be a time of heightened caries activity and periodontal disease. Oral health-related behaviors can be modified to improve oral health status. The family networks and the built environment can promote or inhibit health behaviors. The aim of this study is to implement and evaluate a behavior theory-based, integrated family intervention via mobile messaging to improve oral health of adolescents.

**Methods:**

This is a three-arm parallel-design cluster-randomized controlled trial. This trial will allocate 12 local secondary schools (clusters) in Hong Kong to three test or comparison groups with a ratio 1:1:1. The enrolled Form II to IV students (ages 12 to 15) will be eligible for participation. The intervention to three study groups will be (i) Health Belief Model (HBM)-based mobile messaging to the adolescents and their parents, which will consist of several blocks of HBM-based messages and reinforcement during 24 weeks; (ii) same HBM-based messaging to adolescents only; and (iii) delivering e-version of oral health education pamphlets to adolescents. The primary outcome will be caries increment 2 years post-intervention. Changes in oral health self-efficacy and behaviors, oral hygiene, and gingival status will be the secondary outcomes.

**Discussion:**

No school dental care service is available to secondary school students in Hong Kong. This study will be the first to test a theory-driven and family-engaged preventive intervention among adolescents in Hong Kong. Findings will contribute to developing a low-cost, feasible, and efficient oral health preventive program for adolescents.

**Trial registration:**

ClinicalTrials.govNCT05448664. Registered on 7 July 2022.

## Administrative information

Note: the numbers in curly brackets in this protocol refer to SPIRIT checklist item numbers. The order of the items has been modified to group similar items (see http://www.equator-network.org/reporting-guidelines/spirit-2013-statement-defining-standard-protocol-items-for-clinical-trials/).

**Table:** 

Title {1}	Family behavior theory-based intervention via mobile messaging to improve oral health of adolescents: study protocol for a cluster randomized controlled trial
Trial registration {2a and 2b}	ClinicalTrials.gov NCT05448664. Registered on 7 July 2022. All items from the World Health Organization Trial Registration Data Set can be found in the present protocol.
Protocol version {3}	Version 1.0_ dated Dec.30, 2021
Funding {4}	Health and Medical Research Fund (Reference no: 19201281), Food and Health Bureau, Government of Hong Kong SAR, China
Author details {5a}	Affiliations: - PL, MCMW, and ECML : Applied Oral Sciences & Community Dental Care, Faculty of Dentistry, The University of Hong Kong; - GHML and CKYY: Paediatric Dentistry, Faculty of Dentistry, The University of Hong Kong
Name and contact information for the trial sponsor {5b}	Food and Health Bureau, Government of Hong Kong SAR Email Address: enquiry@fhb.gov.hk Office Telephone: (852) 3509 8765 Fax: (852) 2541 3352
Role of sponsor {5c}	The funding body plays no role in the design of the study and collection, analysis, and interpretation of data.

## Introduction

### Background and rationale {6a}

Adolescents are very broadly defined as youths between the ages of 10 to 19 [1]. Due to some unique physical, social and psychological features in the adolescent population, adolescence can be a time of heightened caries activity and periodontal disease due to an increased intake of cariogenic substances (e.g., consumption of high quantities of refined carbohydrates and sugar-containing beverages) and inattentive to proper oral hygiene procedures due to a low priority [2].

As dental caries and periodontal disease are highly determined by one’s “lifestyle” and are preventable, primary prevention by addressing risk and protective factors and improving oral health behaviors is key to reversing the unhealthy trends. Since adolescents is a critical transitional stage to adopt positive health habits, efficient interventions at this age are essential for preventing oral diseases over a lifetime.

Traditional oral health education (OHE) focuses on solely the dissemination of information and normative advice, which may not result in changes in the recipients’ behaviors [3]. Over the years, behavior theory-driven approaches have been developed and demonstrated potential in improving oral health knowledge, attitudes and behaviors, and caries prevention in adolescents [4]. Health Belief Model (HBM) has been recommended as a useful theoretical model to explain and promote healthy behaviors [5]. HBM suggests that health-promoting behaviors can be triggered by six domains: perceived susceptibility, perceived severity, perceived barriers, perceived benefit, cue to action, and perceived self-efficacy. HBM-based intervention, to some degree, has been introduced to the field of dentistry. Some studies indicated that face-to-face HBM-based education has a significant effect in improving oral health behavior for school children and adolescents [6, 7].

Nevertheless, face-to-face HBM-based intervention can be resource-intensive and challenging to implement at the population level. It may require participants’ frequent on-site participation which could bring to low adherence and high dropout rates. A more convenient, accessible, and time-efficient approach is needed and preferred. Mobile health (mHealth) is a rapidly expanding field in the digital health sector, providing healthcare support, delivery, and intervention via mobile technologies such as smartphones, tablets, and wearables. A key benefit of mHealth is that it has the potential to deliver personalized, interactive, and adaptive health interventions in the context of an individual’s everyday life. It could overcome the traditional barriers faced by healthcare practitioners (such as time and resource constraints), especially among the hard-to-reach populations [8]. To date, there is some evidence that behavior theory-based oral health promotion utilizing mobile messaging app was effective in improving oral hygiene among adolescents with fixed orthodontic appliances [9, 10]. Whereas the use of social media-based applications alone, without a behavior theory-based model, failed to change oral hygiene behavior [11].

It has been reported family network and the built environment may work together to promote or inhibit lifestyle behaviors. Parents have a central role in educating their children and encouraging and supporting their healthy lifestyle choices [12]. Current concepts of oral health education acknowledge the importance of involvement at individual, family, and even community levels [4, 13]. However, studies on how behavior theory-based intervention can be used in the family network and the built environment to improve adolescent oral health are very rare.

To sum up, inspired by HBM combined with a family network approach and using mobile technology as a vehicle, a viable oral health promotion model will be explored and tested in this study. Its effectiveness in promoting the oral health of adolescents will be evaluated in a randomized controlled trial setting.

### Objectives {7}

The objective of this study is to investigate the effectiveness of the family- and HBM-based mobile-health behavioral intervention in enhancing adolescents’ good oral health behaviors (mainly oral hygiene practice and free sugar intake control) and preventing common oral diseases (dental caries and periodontal diseases).

The research hypothesis is the proposed family- and HBM-based mobile-health behavioral intervention is more effective than intervention on the adolescents alone or prevailing OHE in improving the adolescents’ behaviors and oral health.

### Trial design {8}

This study is a three-arm parallel design, superiority, cluster-randomized controlled trial comparing three interventions. Interventions will be applied at the cluster (school) level. Either active or comparator intervention is administered to the school by randomization to prevent contamination. The allocation ratio is 1:1:1.

## Methods: participants, interventions, and outcomes

### Study setting {9}

This study will be carried out in 12 secondary schools in Hong Kong, the primary sampling unit, and formed the clusters of the trial. A list of secondary schools will be obtained from Hong Kong Education Bureau (https://www.edb.gov.hk/en/student-parents/sch-info/sch-search/schlist-bydistrict/index.html). To balance the social-economic status, 3 local co-educational public or government-subsidized schools from Hong Kong Island, 6 from Kowloon, and 3 from New Territories will be selected and randomly allocated to the 3 groups within each geographical region of Hong Kong.

### Eligibility criteria {10}

The target population will be adolescents aged 12 to 15 years old (Form II to Form IV students) and their parents or primary caregivers. The Form-I students will not be invited because they attended the School Dental Care Services provided to primary school students by the Department of Health less than one year ago.

Inclusion criteria are as follows: (i) Chinese ethnicity; (ii) student living with their parent(s) or primary caregiver(s); and (iii) both student and parent(s) or primary caregiver having their access to a personal mobile phone with certain Apps to receive the messages in time (e.g., WhatsApp or WeChat).

Exclusion criteria are as follows: (i) student currently on a special diet (e.g., severe inflammatory bowel disease); (ii) student has medical conditions known to affect growth or eating (e.g., diabetes, cystic fibrosis); and (iii) enrollment in other oral health promotion programs or research studies.

### Who will take informed consent? {26a}

The participating secondary schools will help to invite students and their parents/caregivers. One class in each Form II to Form IV student will be approached. The information sheet and consent form will be distributed to the students and their parents via school. The parent/caregiver and the research investigator will sign the informed consent.

### Additional consent provisions for collection and use of participant data and biological specimens {26b}

An ancillary study that uses participants’ dental plaque and/or saliva to explore the oral microbiome composition will be proposed for selected participants. Additional consent will be provided for the collection and use of participant biological data.

### Explanation for the choice of comparators {6b}

This study adopts two active control groups to verify the effectiveness of the behavioral theory-based family intervention via mobile messaging for oral health promotion. One of the active control groups will receive similar mobile messages as the test group does not include their parents. The other active control group will receive the e-version of OHE pamphlets published by the government, which could be considered the prevailing OHE.

### Intervention description {11a}

#### Group (i) family- and HBM-based behavioral intervention via mobile messaging

The mobile messages based on the HBM model will be sent to the students and their parents in two stages over 24 weeks.

##### Stage I — HBM message series

During the 1st week to the 12th week, text messages exchanged with participants will be organized into six domains guided by HBM [14]. Each domain will comprise various topics and types of messages, e.g., text, photos, or animation demonstration on toothbrushing and flossing. All the messages will be developed using evidence-based content from previous research findings. First, the students and their parents must have some basic knowledge of dental caries and periodontal diseases so that they can be motivated toward a healthy dentition for adolescents (perceived susceptibility). Second, they must perceive that adolescents with poor oral health care and highly sugary diet are at increased risk for oral disease and that poor oral health could also affect general health (perceived severity). Third, the students and parents must also be convinced that regular toothbrushing and control of sugar intake for their children are effective to prevent oral diseases (perceived benefits). Fourth, the students and their parents will identify the barriers to daily oral hygiene practice and added-sugar intake control (perceived barriers). Fifth, the presence of an internal or external stimulus, referred to as “cue to action,” triggers the student’s self-oral health care behavior and promotes the parents to involve, support, and monitor the daily oral health behavior of their children. The toothbrushing and sugar intake control reminders and tips will be designed to support behavior change (e.g., “Time to brush your teeth.” and “Remember to bring along your water bottle so you possibly will not go for a sweet beverage after sporting.”). Finally, empower the student’s self-efficacy through the interaction between researchers and his/her family (perceived self-efficacy). On average, participants are expected to receive a series of 3–5 messages every week during these 12 weeks.

The parents will also receive the mobile message to improve the family’s healthy lifestyle choices except for the knowledge regarding adolescent oral health. For example, “Please use tap water for cooking” (tap water in Hong Kong contains 0.5 ppm fluoride and is a benefit for caries prevention) and “Please don’t prepare too many sugar-added snacks for your child.” The parents will also receive reminder messages to observe, supervise and encourage their child’s proper oral health behavior (e.g., “Do you know your child is brushing his/her teeth properly?” etc.).

##### Stage II — feedback and reinforcement

From the 13th week onwards, we will ask the students and their parents about students’ self-efficacy and collect self-reported information on adherence. For example, we will ask the students “How many of the past seven days have you brushed your teeth twice daily?” and “How many of the last seven days have you taken sweet-beverage more than twice daily?”). Messages of assessment and feedback (e.g., “Great job!”) will be feedback to the students and their parents. The participants will expect to receive 1–2 messages every week over these 12 weeks.

#### Group (ii) HBM-based behavioral intervention via mobile messaging to adolescents

Text messaging based on the HBM model and reinforcement same in group (i) will be delivered to the participating students over 24 weeks. The parents will not receive the intervention.

#### Group (iii) Delivering e-version of oral health education pamphlets to adolescents

The e-version of three pamphlets, namely “*Cleaning teeth properly – You can do it*,” “*How to use dental floss*,” and “*Healthy diet, healthy teeth*” published by the Department of Health, Hong Kong SAR (http://www.toothclub.gov.hk/en/en_index.html) will be delivered separately via mobile messaging.

## Criteria for discontinuing or modifying allocated interventions {11b}

Enrollment is voluntary. The participants can withdraw from the study at any time after enrolment without giving any reason. The statement will be clearly stated in the information sheet to the students and their parents before obtaining their consent. The intervention in this study is mobile messaging, with no harm to participant health.

## Strategies to improve adherence to interventions {11c}

To check compliance with the intervention, we will communicate with the participants in the intervention groups every three months through messaging or a short online questionnaire. The pre-set questions of Goal Attainment Scaling (GAS) will be sent to the participants to check their compliance with the intervention [15]. The participants will self-assess their behavioral goal attainment of expected outcomes. It enables a linkage of reports and measures from the participants to upper levels of project management.

## Relevant concomitant care permitted or prohibited during the trial {11d}

During the trial, all the participants will not prohibit from dental visits for check-ups or treatments. The specific category of the dental visit will be asked for and recorded in each follow-up (e.g, dental fillings, teeth cleaning, and orthodontic treatment).

## Provisions for post-trial care {30}

We will provide dental examination reports to the participants, and suggest dental visits when dental problems are found during the trial. There is no additional post-trial dental care for all participants.

## Outcomes {12}

The primary outcome will be dental caries increment (by tooth level). The students will be examined at the schools at baseline, 1-year, and 2-year follow-up. Dental caries assessment will base on the ICDAS criteria (International Caries Detection and Assessment System), which will be used to assess both non-cavitated (codes 1 and 2) and cavitated caries lesions (codes 3–6) [16]. The examiners will examine occlusal, mesial, distal, buccal, and lingual surfaces of each tooth.

The secondary outcomes will be:

i) Gingival status measured by the percentage of sites with gingival bleeding (BOP%), as recommended by the WHO for conducting oral health surveys [1];
ii) Oral hygiene status using the Visible Plaque Index (VPI). The percentage of plaque on the buccal and lingual surfaces of all indicated teeth will be recorded [1];
iii) Average frequency of toothbrushing per day (2 times as preferred);
iv) Average frequency of intake of sugary snacks/drinks per day (2 times or less frequent as preferred);
v) Average amounts of free-sugar intake per day (<25 g as preferred); and
vi) Oral health self-efficacy on behaviors (questionnaire score rated on a 4-point Likert scale, e.g., 1 = not confident at all to 4 = very confident)

## Participant timeline {13}

**Fig. 1:**
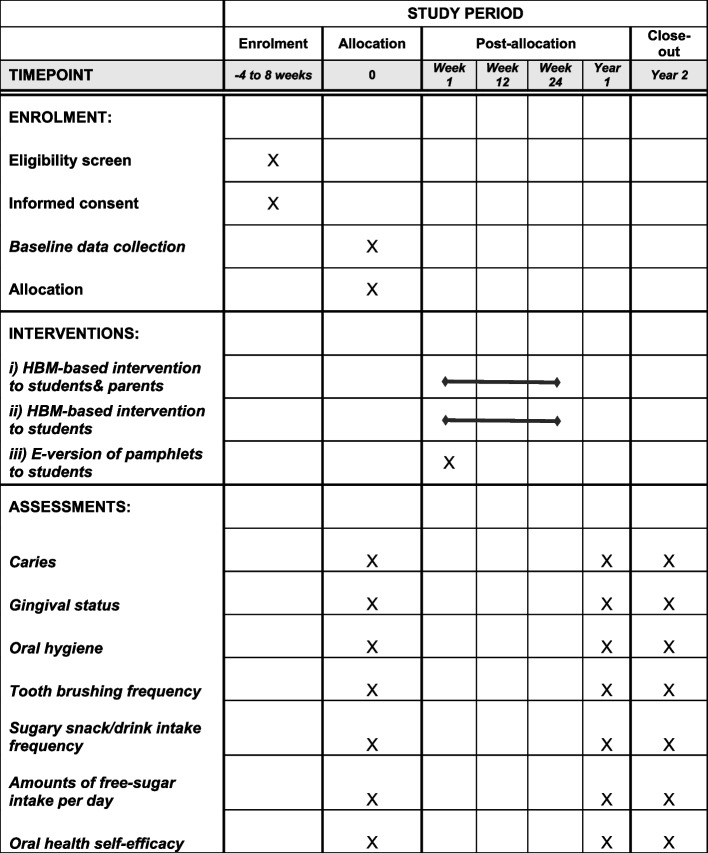
Schedule of enrolment, interventions, and assessments

The participant timeline is shown in Fig. 1. The schematic diagram template was downloaded from http://www.spirit-statement.org/publications-downloads/.

## Sample size {14}

The sample size has been calculated using G*Power software. Our previous study showed that motivational interviewing was more effective than prevailing OHE in preventing dental caries among adolescents [17]. In this proposed study, the sample size is calculated based on mean and standard deviations of the primary outcome measure — the number of new carious teeth (∆DMFT). From the pre-existing study, an effect size of 0.21 was obtained for tooth status over 24-month evaluation (∆DMFT: test group=0.12 vs. control group=0.34; SD ~ 0.50) [18], which could be considered a moderate size convention for 1-way ANOVA and clinically significant for the primary outcome [19]. Based on a significance level of 5% and a targeted statistical power of 80%, 74 participants in each group will be required. Considering a typical value of ICC = 0.03 in a cluster randomized clinical trial setting [20] and if 75 children are to be recruited from each school, the design effect will be 3.22 [21]. Assuming a similar dropout rate as our previous study in secondary school at the 2-year follow-up (dropout rate: 10%) [17], the sample size will be increased to 300 in each group, thus 900 altogether. Then 4 schools in each group, a total of 12 schools will be recruited.

## Recruitment {15}

As we have completed several clinical trials in secondary schools previously, the number of schools for achieving the target sample size will be secured from previous collaborators. Considering the refusal rate was around 20% in our previous studies, one class (class size is around 30 to 40 students) in each Form II, III, and IV will be purposely selected and invited to join the study via school. The sample size of 900 is assumed to achieve by contacting a total of 12 (school)× 3 (grade)× 30~40 (student) = 1080~1440 students initially.

## Assignment of interventions: allocation

### Sequence generation {16a}

Following the recruitment of 12 schools, schools will be randomly allocated to each of the study groups. The allocation sequence will be generated using Microsoft Excel by the project statistician not involved in the data collection.

### Concealment mechanism {16b}

The allocation sequence concealment will be ensured as the statistician will not release the allocation sequence until the baseline data collection has been completed.

### Implementation {16c}

The project research assistant will enroll the participants via the principal of the school. He/she will be informed of the intervention assignments of the study clusters after baseline data collection and then assign participants to interventions and in charge of implementing different interventions correspondingly.

## Assignment of interventions: blinding

### Who will be blinded {17a}

In the present study, the outcome assessors (dental examiners), and data analysts will be blinded to the group assignment of the study participants. Dataset submitted to the data analysts will only contain group numbers whereas the intervention strategy is blinding. Masking of participants would be impossible because the consent procedure required by the Ethics Committee informing participants about the content of the study.

### Procedure for unblinding if needed {17b}

There is no anticipated harm to trial participants so there is no plan for unblinding.

## Data collection and management

### Plans for assessment and collection of outcomes {18a}

The data collection will involve oral examination and questionnaire surveys. Students will have oral examinations at school at baseline, 1-year, and 2-year follow-up by one or two trained, experienced, and calibrated examiner(s). An intra-oral LED light, disposable mouth mirrors, and WHO dental probes will be used during the clinical examination. No x-ray will be taken. Inter-examiner and intra-examiner reliability will be assessed.

Self-completed questionnaires will be delivered to the students at baseline, 6 months, 1-year, and 2-year follow-up through the schools. The items in a validated oral health behavior questionnaire for adolescents based on the health belief model (OHBQAHBM) will be used [22].

Information on the social-demographic backgrounds of the family will also be collected.

### Plans to promote participant retention and complete follow-up {18b}

Follow-ups will be incentivized by offering participants a set of adolescent oral health care products upon completion of each follow-up assessment.

### Data management {19}

All data collection of the clinical charting form will take place physically in the school and will be recorded in hard copy. The questionnaire will be distributed via school, the research team will collect it on the day of the dental checkup. The research assistant will check out the missing data or unreasonable information, and amend the response via phone call or mobile messages.

Data entry and coding will be completed by a research assistant. To ensure accuracy, data proofreading and range checks for data values will be applied by another research assistant. A regular check will be run for data consistency and quality by project investigators.

All the hard copies of the clinical charting form and questionnaire will store in locked cabinets in the Faculty of Dentistry HKU. The dataset will be kept in a secured computer server equipped with a firewall password. The data only can be accessed by the ethics committee, the Institutional Review Board (IRB) of the University of Hong Kong/Hospital Authority Hong Kong West Cluster, and the research team. The participating schools will not obtain and maintain any individual research data.

### Confidentiality {27}

The research data will be handled in line with the Hong Kong Hospital Authority’s policy in handling/storage/destruction of patients’ medical records.

During the trial, a de-identified database in which the student and parent names and contact numbers are eliminated will be for exchange among research team members and analyses. Only the research assistant responsible for the mobile messaging could access the password-protected file with participant identifying information. All the personal data will be destroyed after 3-years of storage.

### Plans for collection, laboratory evaluation, and storage of biological specimens for genetic or molecular analysis in this trial/future use {33}

The biological sample of selected participants will be collected and stored for oral microbiome composition analysis. Unstimulated saliva or/and dental plaque will be collected by a dentist in the study sites and stored in the investigator’s refrigerator at −80°C for future use.

## Statistical methods

### Statistical methods for primary and secondary outcomes {20a}

The effectiveness of HBM-based intervention will be evaluated by comparing the differences in the outcome variables among the three groups. For the primary outcome, linear mixed models will be constructed to compare the dental caries increment and other continuous outcome variables such as BOP% and VPI over time. Socio-demographic variables (gender and parental education level) will be controlled for. Three-level random intercept models will be considered for evaluating the clustering effect and longitudinal data: time as level 1, students as level 2, and schools as level 3 so that the interaction between times and intervention groups would be tested. Multi-level logistic regression adjusting for the effects of possible confounding factors for the clustered data [21] will be performed to test the differences in the prevalence of dental caries, and proportions of other categorical outcome variables such as frequency of toothbrushing and sugar intake among the three groups. The level of statistical significance for all tests will be set at 0.05. All the analyses will be performed using the SPSS software package.

### Interim analyses {21b}

Interim analyses will not be planned in this study. The intervention in this study is mobile messaging with no unacceptable adverse effects, so there is no need to terminate the trial earlier.

### Methods for additional analyses (e.g., subgroup analyses) {20b}

Subgroup analyses will not be applied in this study.

### Methods in analysis to handle protocol non-adherence and any statistical methods to handle missing data {20c}

The non-adherence of study participants will be recorded (e.g., crossing over to the other randomized group or registering another oral health promotion project). The reasons for withdrawal will also be asked and recorded. Intention-to-treat (ITT) approach will be used for data analysis.

In cases with missing data (particularly for the questionnaire), telephone calls or text messages will collect the missing information. If failed, the data imputation will perform using the mean/mode of the variable from the same group of participants if the missing data is less than 20%. If there is missing data in the follow-up, the last observation carried forward method will be adopted. If 20% or more of the data is missing, then the case will be excluded from the data analysis. The sensitivity analyses will perform to see how great is the impact of missing data and how different data imputations may affect results.

### Plans to give access to the full protocol, participant level-data and statistical code {31c}

The protocol will grant access to the DataHub, a library e-platform of The University of Hong Hong when the completion of the study.

## Oversight and monitoring

### Composition of the coordinating center and trial steering committee {5d}

The principal investigator and an expert in dental public health in our research team will be responsible for coordination with different study sites. They will monitor recruitment rate, and follow-up rates and deal with any problems that deviate from the protocol.

The research investigators comprise the trial steering committee, to monitor and supervise the study’s progress toward its interim and overall objectives. Any amendments to the protocol where appropriate, presentations of results, and data release will be consented to by the committee. The funding agency and IRB will be informed about the mentioned actions through principal investigator reporting.

### Composition of the data monitoring committee, its role and reporting structure {21a}

A Data Monitoring Committee (DMC) will not be considered as this study is about low-risk preventive interventions. The principal investigator will be responsible for the data monitoring and complying with the reporting requirements to the funding agency and IRB. The reporting includes the expected and actual recruitment numbers, dropout rates, and any adverse events.

### Adverse event reporting and harms {22}

Adverse events and other unintended effects of trial interventions will be reported to the funding agency and IRB biannually.

### Frequency and plans for auditing trial conduct {23}

The principal investigator will report the progress of the trial conducted regularly and the final research findings to the funding agency and IRB.

### Plans for communicating important protocol amendments to relevant parties (e.g., trial participants, ethical committees) {25}

All the proposed protocol amendments will be discussed and agreed upon by all the investigators, approval will be obtained from both the funding agency and IRB. The information will be updated in the trial registry as well. The trial participants will also be informed and consent.

### Dissemination plans {31a}

Supposing the results are favorable in showing our student participants who received family-integrated HBM-based messages would have better oral health-related behaviors and better oral health outcomes, we would like to disseminate the results to:

i) Department of Health, the Hong Kong Government. The study results will provide much-needed evidence for further developing and implementing a new oral health promotion strategy for adolescents, helpful for the Hong Kong government to re-visit the dental public health policies on adolescents and translate them into practice.
ii) Local dental professional bodies (Hong Kong Dental Association, Hong Kong Society of Pediatric Dentistry) and local medical institutions with pediatricians, medical practitioners, and nurses working in adolescent health care.
iii) Education Bureau and secondary schools. Effective oral health education could be disseminated through school internet platforms. It creates an opportunity for interdisciplinary partnerships with dental professionals and educational professionals, and we hope to explore a feasible and cost-effective strategy by the linkage of schools, students, and families.

By interim and final reporting, the research findings will be presented at international dental conferences and published in international peer-reviewed journals.

## Discussion

The population-based oral health survey in Hong Kong [23] has shown that 23% of 12-year-old children in Hong Kong had caries experience. Although the extent/severity of caries was low (mean DMFT 0.4), most 12-year-old children (86%) had gingivitis, and calculus was prevalent (64%). The lower prevalence of caries among the Hong Kong adolescent population is associated with the School Dental Care Service provided by the Department of Health, which includes a range of preventive and basic dental care for grade 1 to 6 primary school children. However, School Dental Care Service is not available to secondary school students. Additionally, this survey [23] has shown that 32% of the adolescents did not brush their teeth twice per day and that 36% snacked twice per day or more often. We also found that the oral health status worsened from childhood to early adulthood following a cohort of 638 students from 12 to 18 years old in Hong Kong [24]. Hence, there is a strong need to deliver oral health education among this specific age group, highlighting the significance of this proposed project.

For improving oral health in adolescents, this study would be a first step towards the mobile-health-associated Sustainable Development Goals by the WHO [25], as there are no mHealth-driven oral health activities among adolescents in Hong Kong. For there is no governmental oral health service to secondary school students, we hope such a simple intervention using modern-day technology may facilitate positive health behaviors among adolescents, which benefit them for a lifetime.

The innovation of this trial is the combination strategies which is still controversial about the effectiveness of two levels of influence in socio-ecological models (individual and family). Besides, the intervention is theoretically informed, drawing from a behavioral theory from the social-psychological field.

The limitation of this study is the person who can receive the message via smartphone will be recruited, without covering the poor communities with no smartphones. There is a concern about generalizability. Besides, the participants are limited to the cohort of youth in school, therefore the outcomes could not be generalized to the out-of-school adolescents as there could be potential peer effects in our study participants.

The potential pitfall in this proposed study would be that dental examinations could not be conducted at schools due to the COVID-19 pandemic. The participants will have a follow-up at the teaching hospital of the Faculty of Dentistry, The University of Hong Kong. This situation may result in a higher rate of non-attendance and decrease the power of the study. We currently estimate the drop-out rate as 10% for the outcome. To make sure enough sample size is reached, we will assess the attendance rates at the dental hospital for all groups as early as practicable to give a reasonable estimation, and try to arrange future dental examinations in the schools when possible depending on the COVID-19 situation at that time.

## Trial status

ClinicalTrials.gov Identifier: NCT05448664. First posted and registered on 7 July 2022. Recruitment will begin in December 2022 and end in June 2023. The project is anticipated to end in June 2025.

## Data Availability

The final trial dataset will be uploaded to DataHub, the University of Hong Kong for public access by the completion of the study.

## Abbreviations

AAPD: American Academy on Pediatric Dentistry
BOP: Bleeding on periodontal Probing
DMC: Data Monitoring Committee
DMFT: Decayed, Missing due to caries, and Filled Teeth in the permanent teeth
GAS: Goal Attainment Scaling
HBM: Health Belief Model
ICC: Intracluster Correlation Coefficient
ICDAS: International Caries Detection and Assessment System
IRB: Institutional Review Board
ITT: Intention-to-treat
mHealth: mobile Health
OHBQAHBM: Oral Health Behavior Questionnaire for Adolescents based on Health Belief Model
OHE: Oral Health Education
VPI: Visible Plaque Index
WHO: World Health Organization
